# Fabric Coated with Shape Memory Polyurethane and Its Properties

**DOI:** 10.3390/polym10060681

**Published:** 2018-06-19

**Authors:** Md Anwar Jahid, Jinlian Hu, KwanHa Wong, You Wu, Yong Zhu, Hogan Hong Sheng Luo, Deng Zhongmin

**Affiliations:** 1 Institute of Textile and Clothing, The Hong Kong Polytechnic University, Hung Hom, Kowloon, Hong Kong 999077, China; mdjahid09@gmail.com (M.A.J.); kwanha.wong@connect.polyu.hk (K.H.W.); mr.chris-wu@connect.polyu.hk (Y.W.); 2 Shanghai Hiend Polyurethane, Inc., Jinshan District, Shanghai 201500, China; frankzhu@nami.org.hk; 3 Faculty of Chemical Engineering and Light Industry, Guangdong University of Technology, Guangzhou 510006, China; hongshengluo@163.com; 4 School of Textile Science and Engineering, Wuhan Textile University, Wuhan 430200, China; zhongmindeng163@gmail.com

**Keywords:** shape memory polymer, water resistance, water vapor transmission, cotton fabric, coating

## Abstract

In order to provide thermo-physiological comfort to the wearer, textile materials should have some functional property like water-resistance or water vapor transmission (WVT), so that perspiration can evaporate and be transmitted from the body surface to the environment even in extreme weather conditions that require wind and rain proof. Shape memory polyurethane (SMPU) could possibly be a candidate; it is a functional textile material that meets such requirements. In this research, we synthesized a series of SMPUs, which are responsive materials, to test whether these coated cotton fabrics could provide thermal insulation with lower permeability at low temperature or low relative humidity (RH), and high permeability at room temperature or above, or high relative humidity with its water-resistance property. In this paper, we have proposed a segmented shape memory polyurethane for coated textiles in order to have a water-resistant textile with good water vapor transmitting ability.

## 1. Introduction 

Comfort is a satisfying state of physical and emotional harmony between the human being and the environment atmosphere [1,2,3]. Water resistance and water vapor transmission (WVT) through textiles has a great effect on the thermo-physiological comfort of the human body. Thermal comfort is maintained by perspiring in vapor form and transmitting it from inside the garment to the outside environment [4]. When the heat transmission from the clothing inside (skin) to the environment decreases, the sweat glands begin to produce liquid perspiration as well. The perspiration in vapor form is known as insensible perspiration and the perspiration in liquid form is known as sensible perspiration [5,6]. When the vapor perspiration is transported to the outside atmosphere it carries body heat, thus reducing or maintaining the body temperature. Body temperature can influence the skin temperature [7]. Therefore, the garment being worn must allow the vapor perspiration to pass through; otherwise, it will cause discomfort [8]. The level of skin wetness is the main reason affecting the perception of discomfort. During sweating, if a garment’s water vapor transfer rate is slow or limited, the relative humidity levels of the garment inside the microclimate will overwhelming increase the evaporation of perspiration. This will increase body temperature and cause heat stress [9]. Water vapor transmission through textile fabrics plays a major role in maintaining the wearer’s body comfort, both in hot and cold weather and during normal and high activity levels [10]. Hence, a clear consideration of the role of water vapor transmission through clothing in relation to body comfort is important for designing high-performance fabrics for specific end applications. To provide wearing comfort to the wearer, textile materials should have a high-capacity of water vapor transmission so that perspiration can evaporate and be transmitted from the body surface to the environment. Shape memory polyurethane (SMPUs) could possibly be the next candidate of smart textile material in the coming decade [11,12].

The contact angle is defined as the angle formed by the intersection of the liquid-solid interface and the liquid-vapor interface. The SMPUs coated fabrics are expected to improve water resistance to a certain extent. Therefore, SMPUs coated cotton fabrics should have low wettability so that the coated fabrics may bead up the water droplets and drip on the surface of the garment. This also indicates a certain level of hydrophobicity of the coated fabric. The lower the contact angle, the greater the wettability of the fabric. When the contact angle equal to 0°, the complete wetting occurs. In contrast, fabric with large contact angles is represented with lower wettability but higher hydrophobicity. When the contact angle θ = 0 or cosθ = 1, complete wetting occurs. When the contact angle is greater than 90° it generally means that the surface of the sample is water resistant. For super hydrophobic surfaces, the contact angles are generally greater than 150°. This means almost no contact between the liquid droplet and the solid surface. The contact angle is closely related to wettability and surface energy [13,14]. 

The coating is one of the important techniques for adding value to textiles. Coating extends the range of the functional performance properties of textiles and the use of these techniques is increasing rapidly as the application for functional textiles become extensive. The coating technique imparts smart properties to fabrics. Having widespread applications across a range of technical textile sectors increases functionality and durability as well as value. 

SMPU is a new functional material that is continuously improving the aesthetic as well as the comfort of the products created from it. It has attracted greater attention in recent years [15,16,17]. A responsive material is a material that changes in shape as a result of a change in temperature/RH; SMPU is a class of polyurethane (PU) that is different from conventional polyurethane in that it is known to originate from the phase-separated structure between hard and soft segments, and the reversible phase transformation of the soft segment. The use of SMPU in clothing textiles is a new concept. In SMPU, the soft segment is responsible for behavior changes according to temperature and humidity and allows the water vapor to pass through [18,19]. When shape memory polyurethane is laminated/coated with a fabric [20,21,22], a smart fabric is formed. Its permeability changes as the wearer’s environment and body temperature change to form an ideal combination of thermal insulation and vapor permeability for army clothing [23,24,25,26]. When the body temperature is low, the fabric remains less permeable and keeps the body heat. When the body is in a sweat condition, it allows the water vapor to escape into the air because its moisture permeability becomes very high with increasing body temperature [27,28,29,30]. It can also keep our body warm by resisting water during rain. 

In this work we have produced a coated fabric with water resistance and permeability properties. Such a coated fabric can be used during rainy weather to protect the body from the rain or some other extreme condition, when both water resistance and permeability are required. In this study, three different types of polyurethane were synthesized and coated on to cotton fabric surfaces. We then investigated the water resistance and water vapor transmission property of the coated fabrics. 

## 2. Material and Method 

### 2.1. SMPU Synthesis 

**Figure 1:**
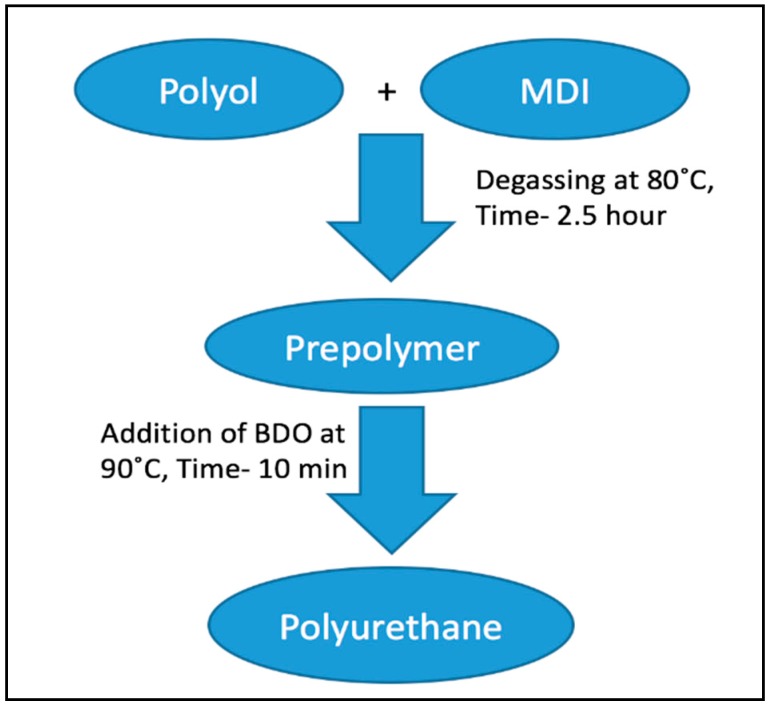
Synthesis procedure of polyurethane.

In this study, three different types of polyurethanes were synthesized for cotton fabric coatings, they are Polyethylene Glycol (PEG), Polytetramethylene Glycol (PTMG), and Polycaprolactone (PCL) based polyurethane. We have used three different types of polyol (PEG, PTMG, and PCL), with a molecular weight of 2000 g/moL. As a di-isocyanate we have used Methylene Diphenyl Isocyanate (MDI) with a molecular weight of 250 g/moL, and 1,4-Butanediol (BDO) with a molecular weight of 90 g/moL as a chain extender. Concentration and synthesis are detailed in Table 1 and Figure 1, respectively.

### 2.2. Sample Preparation 

Scoured, bleached, and dyed 100% cotton fabrics were used in a twill weaving structure, then the fabric samples were cut into 200 mm × 200 mm sizes before coating. The twill fabric we have used is 115 gms/sq.mt, 2/2 twill fabric, so that the twill line can be seen from both sides of the fabric. Cotton fabrics were then coated by a knife over roller machine (Werner Mathis AG, Oberhasli, Switzerland). The coating roller speed was 0.2–0.5 m/min and the linear pressure was set to a 400 daN/cm (dekanewton/centimeter) maximum and applied in a single layer. The thicknesses of the uncoated and coated fabrics were 0.2 mm and 0.30–0.33 mm (Table 2), respectively, and measured by ETOPOO GL-65-75 thickness gauge (accuracy ±0.003 mm). In order to easily identify the coated and uncoated fabrics, the uncoated and coated fabrics were coded and tabulated in Table 2.

The solution of the polyurethane has been added on to the fabric surface. We kept the same add-on percentage for all the fabric. The add-on % is the weight of the polyurethane solution that has been added on to the fabric surface and controls the thickness and evenness of the sample. The add-on % on the coated cotton fabric was about 20% and calculated using the following formula

**Figure 2:**
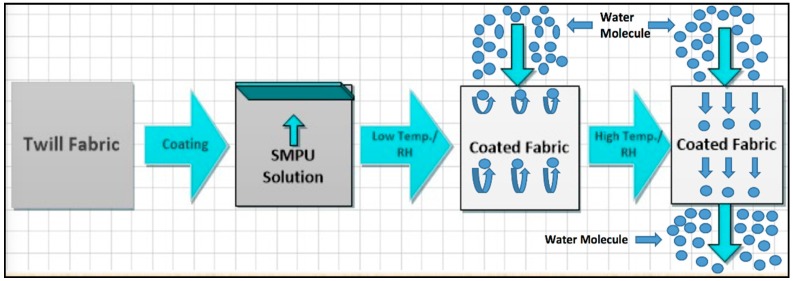
Theoretical approach of fabric coating and water vapor transmission.

Add-on (%) = [(*Y* − *X*)/*X*] × 100 (1)

where *X* is the weight of the uncoated fabric and *Y* is the coated fabric. Figure 2 shows the theoretical approach of fabric coating and water vapor transmission. 

### 2.3. Characterization 

**Figure 3:**
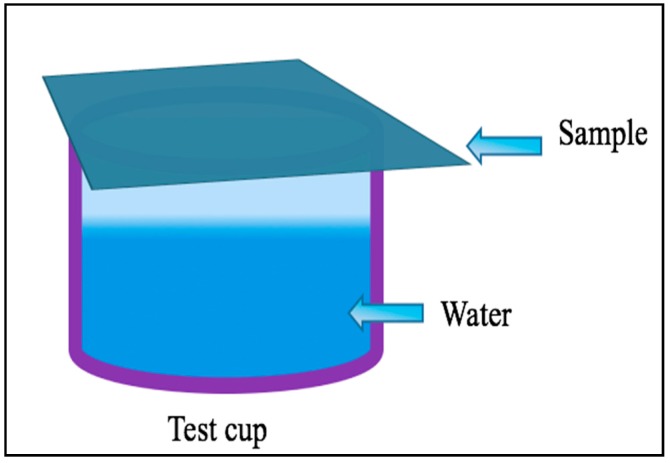
Testing method of water vapor transmission (WVT).

The WVT of the uncoated and coated fabrics was measured according to the ASTM E96 B testing method by using Haida International Equipment (Model-HD-E702-100-4, Dongguan, China), it is a temperature and relative humidity controlled climate chamber where the water vapor transmission test was conducted. The test cup was half filled with distilled water (room temperature (23 °C)) then the film was fixed on the top of the cup and the initial weight was taken before the test cup was placed into the test chamber for 4 h. The final weight was taken after 4 h (convert it to 24 h) to calculate the WVT rate. Figure 3 shows the test method.

The result of the water vapor transmission can be calculated by the following formula

WVT = *G*/*tA* (2)

where *G* is the weight change in grams; *t* is the duration of the test time in day (d) and *A* is the test area in m^2^. Unit—g·m^−2^·d^−1^.

The surface of coated and uncoated fabric was observed by scanning electron microscope (SEM) with (TESCAN VEGA3, Brno, Czech Republic). Two 2 mm samples were cut from two different regions of the sample and then fixed in a carbon tape for gold coating then placed into the SEM machine for analysis and capturing the microscopic image. 

Tensile properties (warp direction) were measured by using an Instron 4411 (Boston, MA, USA) according to ASTM D-2256. The samples were cut into 100 mm × 10 mm (*L* × *W*) squares. By using this technique, we have measured the maximum elongation and tensile strength of the sample at room temperature (25 °C).

Differential scanning calorimetry (DSC) was conducted using a PerkinElmer DSC 8000 (Waltham, MA, USA). 5 mg samples were cut and sealed in aluminum pans and loaded into the DSC chamber for analyzing the Tm of the sample. The unit of heat flow is mW.

Thermogravimetric analysis (TGA) was carried out under the air atmosphere (50 mL/min) on a TG analyzer, Model METLER TOLEDO (Columbus, OH, USA), with a sample weight of approximately 5 mg. The heating rate of 10 °C/min was used in the analysis. The heating range was 50 to 700 °C.

Shape memory properties of the polyurethane were measured using an Instron 5566 (Canton, MA, USA). The shape memory property was measured under the condition of heating the sample at 60 °C for 10 min then elongating it up to 100% and holding it for 10 min, then cooling it at room temperature. The number of cycles was four and the size of the sample was 8 cm × 1 cm. The shape fixity and shape recovery ratio were calculated from the below equations (*ε_u_*: fixed strain; *ε_m_*: maximum strain; *ε_p_*: plastic or residual strain).

(3) $$\mathrm{Shape}\;\mathrm{fixity}\;\left(\%\right)=\;\frac{\varepsilon_{u}}{\varepsilon_{m}}\times 100$$

(4) $$\mathrm{Shape}\;\mathrm{recovery}\;\left(\%\right)=\frac{\left(\varepsilon_{m}-\varepsilon_{p}\right)}{\varepsilon_{m}}\times 100$$

The contact angle of both coated and uncoated fabrics were measured according to ASTM D7334 to investigate the water resistance property of the sample, the contact angle was measured by using tantec contact angle meter (Lunderskov, Denmark). The specimen was placed in the instrument and a drop of de-ionized water placed on the surface using a syringe at room temperature. The contact angle was determined graphically. 

## 3. Result and Discussion 

### 3.1. Water Resistance/Contact Angle 

**Figure 4:**
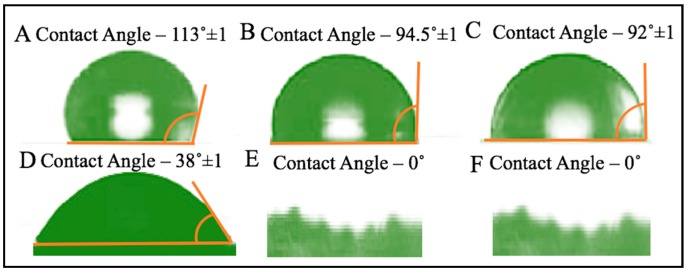
Contact Angle of coated and uncoated fabric. (**A**) Coated after 0 s; (**B**) Coated after 20 s; (**C**) Coated after 2 min; (**D**) Uncoated after 0 s and (**E**) Uncoated after 20 s, and (**F**) Uncoated after 2 min.

**Figure 5:**
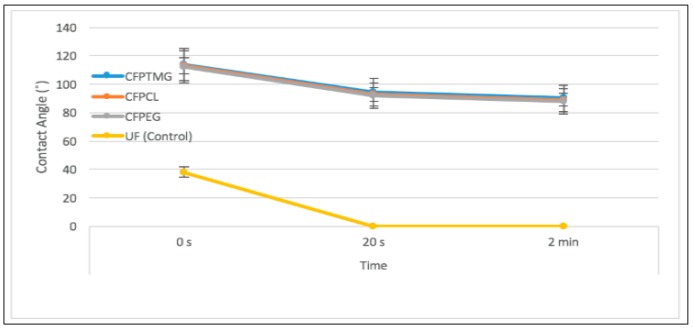
The contact angle of CFPTMG, CFPCL, CFPEG and uncoated with time variation.

**Figure 6:**
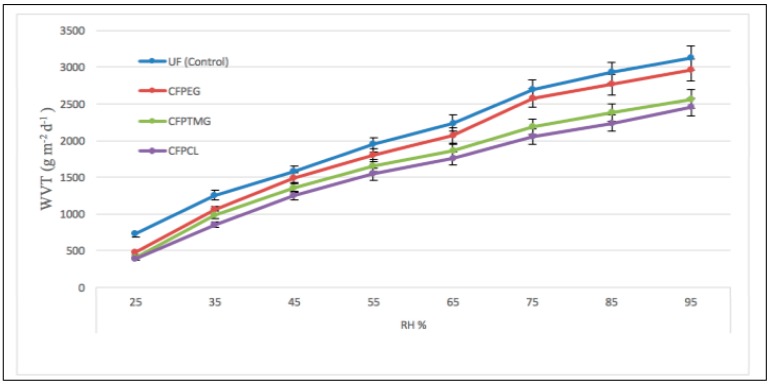
WVT of UF (control), CFPEG, CFPTMG, and CFPCL.

**Figure 7:**
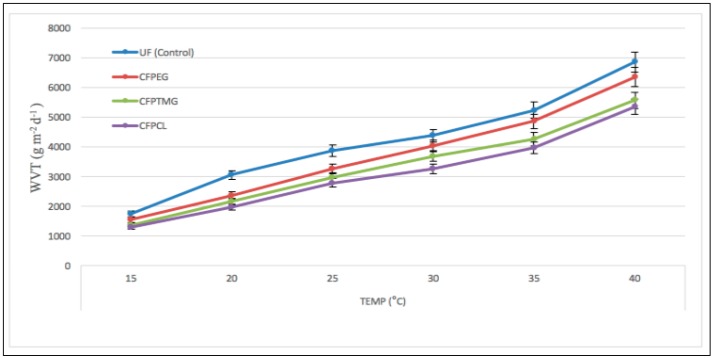
WVT of UF (control), CFPEG, CFPTMG, and CFPCL.

The wettability and water resistance property of the coated and uncoated fabrics were measured by the contact angle measurement of a water droplet at its sample surface (Figure 4). In Figure 4A–C, the water contact angle changes from 113° ± 1 to 92° ± 1, implying the change of the contact angle from good water resistance to water resistance. In Figure 4D–F, the water contact angle changes from 38° ± 1 to 0°, implying the change of the contact angle from slightly wetted to completely wetted. As we can see in Figure 4E,F, the surface is not so smooth because there are some protruding fibers that absorb water. From Figure 4E, we can see that the contact angle of the uncoated fabric after 20 s is 0°, which means it is wetted completely, and the contact angle of CFPEG, CFPTMG, and CFPCL is 94.5° ± 1 (Figure 4B), and after 2 min the contact angle of the coated sample is 92° ± 1 (Figure 4C), which means still water-resistant. However, after a drop of de-ionized water was placed on the sample surface, we immediately observed the contact angle and found that the uncoated fabric was slightly wetted and the coated fabrics were water-resistant. From the result of the contact angle of our experimented sample, we can say that our coated samples are water-resistant where the uncoated sample is not. Figure 5 shows the contact angle of CFPTMG, CFPCL, CFPEG and uncoated with time variation. The polyurethane we synthesized easily passes the water molecule (water vapor transmission result are in Figure 6 and Figure 7) but it can resist the water droplet (normally the size of the water molecule is about 2.75 Å where the size of the water droplet is about 2 mm). The contact angle is mainly dependent on the cohesive forces of the molecule (surface tension) of the surface of the polymer. The coated polyurethane has enough surface energy to keep liquid together, meaning droplet spread-out will not occur.

### 3.2. Water Vapor Transmission of Coated Fabric

The water vapor transmitting ability of UF (Control), CFPEG, CFPTMG and CFPCL are plotted in Figure 6 and Figure 7. The water vapor transmitting ability of the coated fabric was measured, in order to compare the water vapor transmitting ability of coated fabric with different polymeric materials. The results of the water vapor transmitting ability of UF (control fabric) and the coated fabric CFPEG, CFPTMG, and CFPCL are shown in Figure 6 and Figure 7.

As shown in Figure 6, we can see that the WVT for the uncoated fabric increased linearly with the increase of relative humidity (constant temperature (20 °C)) and the WVT of the CFPEG coated fabric shows a better WVT than the all coated fabric during the whole period. Figure 6 shows that the WVT of the CFPTMG and CFPCL coated fabrics was always lower than the CFPEG and the uncoated fabric. The WVT of all coated fabric was similar at the beginning; however, the WVT of all coated fabric increased significantly when enhancing the RH %. The slope of all coated fabrics was so similar that they raised steadily at RH 25% to 65%, but CFPEG increased significantly at RH 65% to 95%. CFPEG has showed significantly better water vapor transmission compared with CFPTMG and CFPCL after RH 65%. As we can see from Figure 6, CFPEG increased significantly after RH 65% and nearly touches the uncoated fabric. The WVT increases with such a high relative humidity because of the matrix between the relative humidity, constant temperature (20 °C) in the chamber, room temperature water (23 °C), and the air velocity in the chamber 0.3 m/s. 

The results of the water vapor transmitting ability of the uncoated (control fabric) and the coated fabric, CFPEG, CFPTMG, and CFPCL against temperature are shown in Figure 7.

As shown in Figure 7, we can see that the WVT for the uncoated and coated fabrics increased gradually with the increase of temperature from 15 to 40 °C (constant relative humidity (70%)). It shows that the WVT of the CFPEG coated fabric was similar to the uncoated fabric throughout the whole period. Figure 7 shows that the CFPEG is very near to the uncoated fabric after 30 °C. After 25 °C, CFPEG increases significantly compared with CFPTMG and CFPCL. Sinem et al. [13] tried to improve the breathability of the coated fabric via micro-cracking and tested the water vapor permeability of the coated fabric according to EN ISO 11092, but their result reveals that the water vapor permeability did not improve enough; from their results the coated sample can pass around 12% of water, where the uncoated sample can pass around 35% of water from the test cup, which means the uncoated fabric is much more water vapor permeable than the coated fabric. The difference between the uncoated and coated fabric is too high. In contrast, the water vapor permeability property of our coated fabric is very near to the uncoated fabric. Our coated fabric is able to transmit water vapor more efficiently because of its soft and hard segment ratio, and the molecular weight of the polyols. 

The effect of temperature or RH on the water vapor transmitting ability of CFPEG, CFPTMG, and CFPCL is highly related by their hard and soft segment ratio and their –OH group. The CFPTMG and CFPCL molecular structure have less change or movement with the increase of temperature or RH compared with CFPEG, and water vapor transmission largely depends on the water affinity of the functional group of those polymers. As we can see, CFPEG attracts and passes more water vapor molecule when compares with CFPTMG and CFPCL. According to the above results, WVT increased with the increase of temperature and RH %. However, for shape memory polyurethane there is a transaction when temperature or RH is high and the soft segments of the SMPU change their state according to temperature or RH in order to facilitate the passing of water vapor. From Figure 6 and Figure 7 we can see that the CFPEG showed better WVT compared with the CFPCL and CFPTMG because of the number of the –OH group in the structure. WVT depends on a hard and soft segment ratio of the polymer and the number of –OH groups in the structure. As we know from the structure, PEG has more affinity to water molecules compared with PCL and PTMG because of its branched structure and number of –OH groups. 

### 3.3. Mechanism of Water Transmittance and Resistance Property of the Coated Fabric

**Figure 8:**
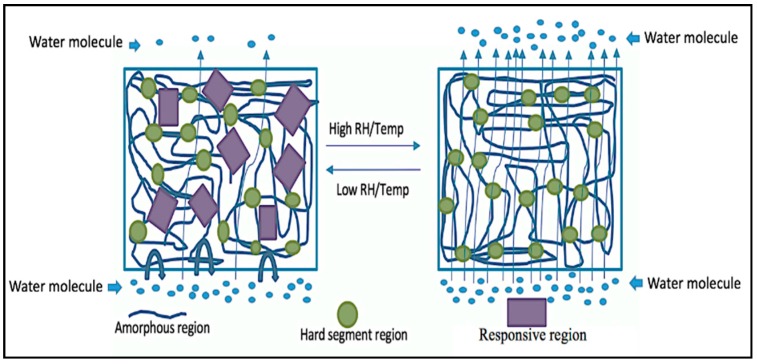
Molecular structure design of responsive polyurethane.

**Figure 9:**
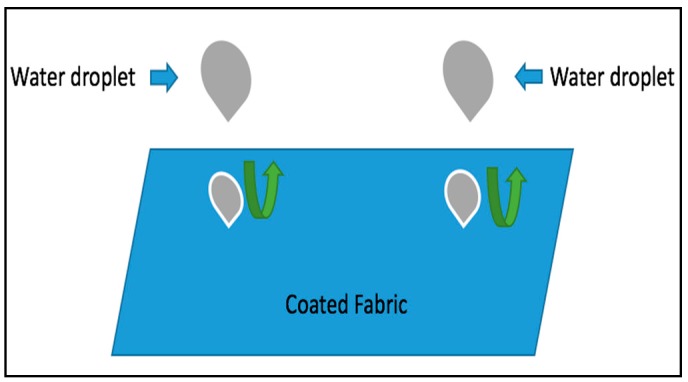
Water resistance of coated fabric.

Non-porous polymeric membranes are a dense polymer; PEG, PCL, and PTMG are all hydrophilic polymers that can absorb and diffuse water molecules and can produce a wicking action that attracts water molecules. A membrane allows the water molecule to transmit. When the water vapor arrives at the surface of the membrane, the membrane absorbs the water molecule because of its hydrophilic nature. The chemical structure and film thickness are the main determinants of transmission ability of the water vapor in a membrane and also the surroundings (Temperature and Relative humidity). In Figure 8 and Figure 9, we explain the factors that influence the water vapor transmission and resistance. Figure 8 reveals that the hydrophilic polymeric membrane can be triggered by temperature and relative humidity, that is why we call our polymer a responsive polymer. Furthermore, permeability occurs through the membrane at the molecular level of sorption-absorption/diffusion-evaporation, therefore, the structure of the polymer plays the main role in controlling the water vapor transmitting ability of the polymer. The polymer interaction and the primary structures of the polymer itself are very important for understanding polymer functions such as sorption, and the permeability of small water molecules. Furthermore, from the above WVT result we can say that fabric coated with PEG has better permeability compared with PCL and PTMG, as we know from the structure that the functional group of PEG is more water absorbent than PCL and PTMG. PEG contains more hydroxylic groups and branched structures that cause the improvement of water molecule passes. 

The water-resistant property of coated fabric was measured and it was found that all the coated fabric is water-resistant while the uncoated one was fully water absorbent. The terms water vapor transmittable and water resistance are seemingly contradictory, but water vapor transmission happens through the interaction of the water molecule and the hydrophilic polyurethane, while water-resistance happens by resisting the water droplet. It is easy for the polyurethane to resist the water droplet because it comes with a number of water molecules together. Figure 8 and Figure 9 show the mechanism of the water vapor transmitting ability and water resistance ability, and the size of the water molecule and the water droplet. As we know, the size of the water droplet is about 2 mm, which is 20 million times higher than the water molecule (the size of the water molecule is about 2.75 Å). Furthermore, normally, the twill structure of the cotton fabric does not have any issue with water vapor transmission, however, it can absorb water very easily (Figure 4). The moisture regain percentage of cotton is about 7.1–8.5%. To avoid the water absorbent property of the cotton fabric we coat them in polyurethane in order to get a water-resistant (rainproof) cotton fabric, and achieve at the same time better water vapor transmission. 

### 3.4. Thermo-Mechanical Properties 

**Figure 10:**
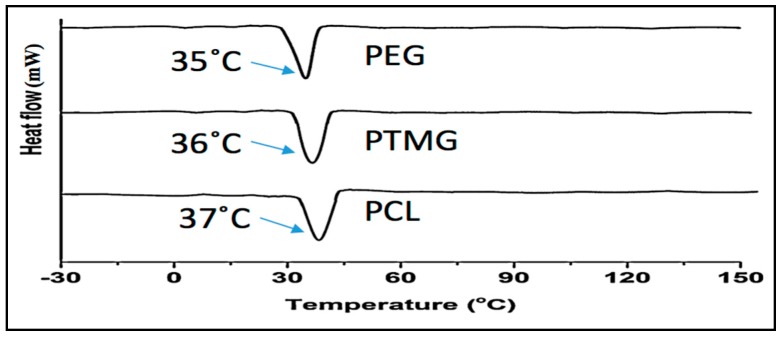
Differential scanning calorimetry (DSC) of different polyurethane.

Shape memory polyurethane is usually a thermally induced process, though it can be triggered by temperature and humidity. Thermally induced SMPUs have the capability of sensing and responding to external stimuli, such as temperature and humidity, in a predetermined way. The polyurethane we have synthesized is segmented polyurethane. It has two different phases (soft and hard). When the temperature or the humidity rises, the soft part of the polyurethane starts to switch its state, which leads to the improvement of WVT with increasing temperature. Figure 10 reveals the melting temperature of the polyurethane. The transition temperature depends on the hard/soft phase ratio and the molecular weight of the polyols. In addition, the crystallinity increases with the increase in soft segment content and molecular weight. As we can see from the Figure 10, the transition temperature of polyurethane is (35–37) °C, which means it can switch in this temperature range. The soft segment influences the water vapor transmission of the polyurethane by the free volume and hydrophilicity of the polyurethane, and it is revealing that the PEG has the lowest transition temperature among these three types of polyurethane. 

**Figure 11:**
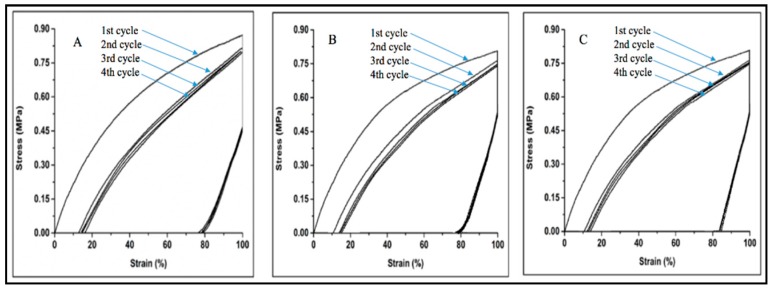
Thermo-mechanical cyclic curves of (**A**) Polyethylene Glycol (PEG); (**B**) Polycaprolactone (PCL); (**C**) Polytetramethylene Glycol (PTMG).

The shape memory property of different polyurethanes are tabulated in Table 3. All the synthesized polyurethane has shown the shape memory property. It can be said that the existence of the irreversible elongation and recovery trend happened as a result of the hard segment network, which gives rise to the ratio of shape recovery and the ratio of fixity shape, which describes the shape memory property of the material. The deformation is normally conducted at a temperature higher than the transition temperature and this deformation can be fixed in the cooling process. Figure 11 shows that when we release the stress of the polyurethane from 0.85 MPA to 0.00, the PEG can keep 100% strain till 0.45 MPA, while PCL and PTMG can keep 100% strain till 0.55 MPA. Then the strain goes down along with stress. As we can see, the strain of PEG, PCL, and PTMG is fixed at 79.4 ± 1.5%, 79.5 ± 1.5%, and 80.1 ± 1.5%, respectively, with zero stress. After cooling it at room temperature we found the shape recovery of PEG, PCL and PTMG are 85.3 ± 1.5%, 86.2 ± 1.5% and 86.7 ± 1.5%, respectively. The plasticity of the polyurethane depends on the shape recovery. We found the plasticity of the polyurethane after deducting the shape recovery from the maximum strain (100%). From these three different types of polyurethane, we can see that the shape fixity and recovery of PTMG is comparatively higher than PCL and PEG, while the fixity and recovery of PTMG is higher because of the crystalline structure of the soft segment. Figure 11 shows the thermo-mechanical cyclic curves of PEG, PCL, and PTMG.

**Figure 12:**
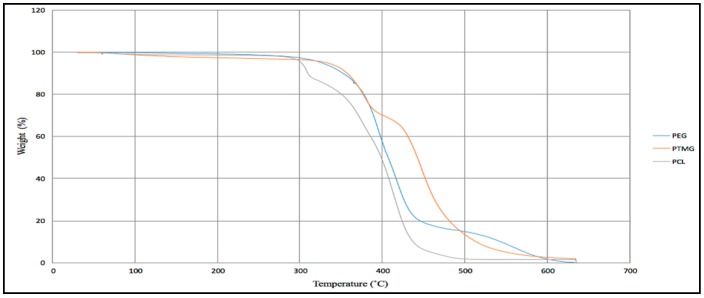
Thermogravimetric curves of three different polyurethane.

Thermogravimetric analysis (TGA) is the method of study of the thermal behavior of the polymer. Figure 12 provides the information about the decomposition of a different kind of polyurethane, and from it we can observe that the decomposition occurs in three steps. According to the first step, the polyurethane showed an initial weight loss (decomposition around 20%) in the range of 50 to 380 °C, which is related to the mass loss of the volatile compound, like some additives which has been used during the synthesis process of polyurethane. The second step is the decomposition of the urethane (around 60% decomposition of the polyurethane) in the range of 380 to 450 °C, and in the third step, the ester groups decompose (around 20% of the polyurethane) in the range of 450 to 650 °C. The thermal stability of these polyurethanes strongly depend on the urethane groups per unit volume. 

### 3.5. Mechanical Properties 

The tensile properties of the SMPU coated fabric were measured to investigate the mechanical strength of the coated fabric. 

The tensile strength of the coated fabrics increased compared with the uncoated control (uncoated) fabrics that have many pores, which would permit sufficient penetration of SMPU to achieve some chemical and mechanical adhesion of the film. The coated fabric is a composite of polymer and fabrics, and the final tensile strength of the polymer coated fabrics is a function of the base fabrics and SMPU materials. Tensile properties and the elongation of the coated fabrics are almost the same. As we can see from Table 4, the elongation and strength of the CFPEG and CFPTMG are similar. The strength of the coated fabric is much better than the uncoated fabric. As we can see from Table 4, the breaking strength of the coated samples is very close and the uncoated one is too low compared with the coated sample, which is the reason the standard deviation for the breaking strength is so large while the standard deviation among the coated samples is 0.58 MPA. 

### 3.6. Scanning Electron Microscopy (SEM)

The surface morphology and properties of the uncoated and coated fabrics were examined by SEM. The cross-section of the above-mentioned specimens was further investigated to determine the changes in the fiber morphology as well as the fabric properties.

**Figure 13:**
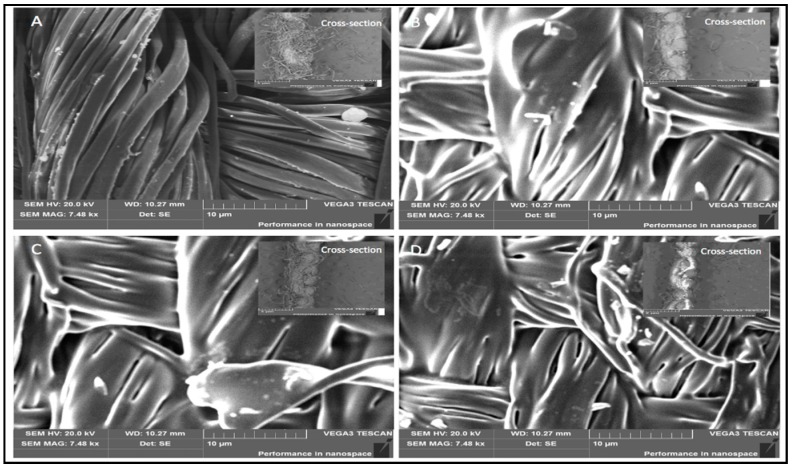
Surface morphology and cross section of (**A**) control fabric (uncoated); (**B**) CFPEG; (**C**) CFPTMG; (**D**) CFPCL coated fabric.

Figure 13A shows the surface morphology of the uncoated fabric. The uncoated fabric surface contained many protruding fibers. The surface was not flat. The cross-section view of the uncoated fabric was not compacted. The cotton fibers were loose in the weaving structure and the fibers were easily eliminated. Figure 13B–D shows the surface morphology of the CFPEG, CFPTMG, and CFPCL. All coating concentrations became flattened compared to the control fabric. The fibers were controlled by the polymer coating. The cross-section view was compacted and thinner when compared with the uncoated one. The cotton fibers were controlled by the coating. 

## 4. Conclusions

The use of SMPU in clothing is a new concept, particularly with water sensitivity (water-resistant and permeable). When SMPU is coated to a fabric, a functional fabric is formed. Its permeability changes as the body temperature and humidity change to form an ideal combination of thermal insulation and vapor transmitting ability for the garment. As we can see from the contact angle, the uncoated cotton fabric is not water-resistant where all the coated fabrics are water-resistant. From the results of the water vapor transmission test, it was observed that the WVT curve of the CFPEG significantly changed when both relative humidity and temperature were altered, whereas the CFPTMG and CFPCL were less sensitive to both the temperature and relative humidity compared with CFPEG. The WVT curve of the CFPEG changed more significantly against both the temperature and relative humidity linearly with the uncoated fabric. It was concluded that the segmented CFPEG is water resistant, thermal, and moisture sensitive. In moisture-sensitive SMPU, the fabric remains less permeable and keeps body heat when the body humidity is low and can resist water droplets when needed. When the body is in a sweat condition, it allows more water vapor molecules to escape into the air because its moisture permeability becomes very high, at the same time it can protect our body from the rain because of its water-resistant property. 

## Figures and Tables

**Table 1:** Concentration details of polyurethane synthesis.

Sample	PEG (Polyol), moL	PTMG (Polyol), moL	PCL (Polyol), moL	MDI (Di-Isocyanate), moL	BDO (Chain Extender), moL	Soft:Hard Segment Ratio
Sample 1	0.082			0.282	0.200	65:35
Sample 2		0.082		0.282	0.200	65:35
Sample 3			0.082	0.282	0.200	65:35

**Table 2:** Coding and thickness of uncoated and different coated fabric.

Sample Code	Description	Thickness (mm)
UF	Uncoated fabric (Control)	0.20 ± 0.01
CFPEG	Fabric coated with PEG	0.30 ± 0.01
CFPTMG	Fabric coated with PTMG	0.32 ± 0.01
CFPCL	Fabric coated with PCL	0.33 ± 0.01

**Table 3:** The shape memory properties of the different polyurethanes.

Polyurethane	Shape Fixity (%)	Shape Recovery (%)	Plasticity (%)
PEG	79.4 ± 1.5	85.3 ± 1.5	14.7 ± 1.5
PTMG	80.1 ± 1.5	86.7 ± 1.5	13.3 ± 1.5
PCL	79.5 ± 1.5	86.2 ± 1.5	13.8 ± 1.5

**Table 4:** Tensile strength and elongation of uncoated and coated fabric.

Sample	Breaking Load (MPA)	Breaking Elongation (%)
UF (Control)	27 ± 1	21 ± 1
CFPEG	36 ± 1	22 ± 1
CFPTMG	36 ± 1	21 ± 1
CFPCL	37 ± 1	20 ± 1
